# Evaluation of pharmacokinetics and relative bioavailability of pentoxifylline and its metabolite in beagle dogs following different formulations

**DOI:** 10.3389/fphar.2024.1488076

**Published:** 2024-11-20

**Authors:** Yuxiang Xu, Hongxin Qie, Haopeng Zhao, Wenlin Gong, Peiyuan Wang, Xiaonan Gao, Jinglin Gao, Zhangying Feng, Mingxia Wang

**Affiliations:** Department of Clinical Pharmacology, The Fourth Hospital of Hebei Medical University, Shijiazhuang, China

**Keywords:** pentoxifylline, sustained-release tablets, enteric-coated tablets, metabolite, pharmacokinetics

## Abstract

A single-oral-dose, two-period cross-over study with a 5-day washout period under fed condition was conducted in six beagle dogs to explore the pharmacokinetic characteristics and relative bioavailability between sustained-release (SR) tablets and enteric-coated (EC) tablets of pentoxifylline (PTX) and its metabolite. The results showed that M5 exhibited the highest exposure level, while M1 demonstrated the lowest in both the SR and EC tablet groups. For PTX and M1, T_1/2_ were 0.42 and 0.55 h, with t_max_ of 1.83 and 1.83 h, respectively, in the SR tablet group; in the EC tablet group, T_1/2_ were 0.38 and 0.47 h, respectively. However, a significantly prolonged absorption process was noted, with t_max_ values of 5.06 and 5.78 h. In contrast, M5 exhibited distinct pharmacokinetic differences compared to PTX and M1. For the SR tablet group, T_1/2_ and t_max_ were recorded at 2.03 and 3.08 h, respectively. In the EC tablet group, T_1/2_ and t_max_ were 1.67 and 5.78 h, respectively. With regard to the geometric least squares mean (LSM) of AUC and C_max_ for SR tablets and EC tablets, the ratios of SR/EC of PTX, M1 and M5 were 67.62% (90% CI, 50.49%–90.55%), 78.18% (90% CI, 54.15%–112.88%), and 119.11% (90% CI, 99.62%–142.41%), respectively, for AUC_(0-t)_. The ratios were 67.62% (90% CI, 50.50%–90.55%), 78.36% (90% CI, 54.48%–112.72%), and 119.39% (90% CI, 100.03%–142.50%) for AUC_(0−∞)_ and 54.36% (90% CI, 36.63%–80.67%), 58.80% (90% CI, 40.84%–84.66%), and 100.51% (90% CI, 89.50%–112.88%) for C_max_, respectively. The AUC ratio predictions of bioconversion results indicated that there was no significant difference in the bioconversion of M1 between the SR tablets and EC tablets, with conversion rates of 0.37 and 0.36, respectively. In contrast, the conversion rate of M5 demonstrated a significant difference (*p < 0.05*) between the SR tablets and EC tablets, with the ratio of 3.09 and 1.91, respectively. Furthermore, the EC tablet group demonstrated notable inter-individual differences and irregular drug absorption, following meals. Consequently, the SR tablets appeared to provide a more stable and controllable therapeutic effect in beagle dogs.

## Introduction

Pentoxifylline (PTX), a derivative of methylxanthine, exhibits significant hemorheological effects and has been utilized for many years to alleviate symptoms of intermittent claudication. Numerous studies indicate that this medication could contribute extensively in enhancing cardiovascular health (McCarty et al., 2016). In addition, PTX is extensively utilized in the adjunctive treatment of ischemic lesions, cardiovascular diseases, alcoholic hepatitis, venous ulcers of the lower limbs, diabetes-related complications, and various other conditions due to its capacity to enhance blood circulation and suppress inflammatory factors (Whitfield et al., 2009; Jull et al., 2012; Bell, 2021; Haque and Mohan, 2003). This drug benefits blood rheology in several complementary ways, including decreasing blood and plasma viscosity, lowering plasma fibrinogen while promoting fibrinolysis, and improving blood filterability by enhancing erythrocyte distensibility and lessening neutrophil activation (McCarty et al., 2016). PTX exerts anti-inflammatory, antioxidant, anti-apoptotic, and anti-fibrotic effects through the regulation of multiple signaling pathways. Its broad application prospects contribute to its widespread use in clinical practice (Mostafa-Hedeab et al., 2022; Khoury et al., 2023).

PTX is extensively metabolized by the liver, undergoes extensive metabolism in humans, resulting in formation of at least seven metabolites (denoted metabolite M1-7), the major circulating ones being M1 [1-(5-hydroxyhexyl)-3,7-dimethylxanthine], M4 [1-(4-carboxybutyl)-3,7- dimethylxanthine], and M5 [1-(3-carboxypropyl)-3,7-dimethylxanthine] (Fantin et al., 2006). The hemorheological and anti-cytokine properties of PTX are known to be retained by racemic (R, S)- M1 and (R)-M1 (lisofylline), the major reductive metabolite (Magnusson et al., 2008). Other research findings indicated that M1 may also play a significant role in anti-fibrosis and the regulation of TNF-α (Peterson et al., 2011). Currently, there were limited studies on the oxidative products M4 and M5 of PTX. Only a few studies showed that M4 and M5 were more potent than PTX in inhibiting neutrophil superoxide anion production, degranulation (lactoferrin release), and surface expression of the β-2 integrin CD11b/CD18 (mac-1). In particular, M5, retaining the modulatory effects of PTX on erythrocyte deformability, platelet aggregation, and neutrophil function but lacking substantial inhibitory activity toward TNF-α production, deserves further preclinical evaluation for its therapeutic potential as a hemorheological and anti-inflammatory agent (Fantin et al., 2006).

Current reports primarily focused on pharmacokinetic studies of the PTX and its main metabolites M1 and M5. The pharmacokinetic result indicated that in healthy volunteers at 2.5 h after administration of a single oral dose of 600 mg PTX, the corresponding concentrations of PTX, M1, and M5 were 175.69, 490.99, and 1061.14 ng/mL, respectively (Vlase et al., 2010). For different indications, including peripheral vascular and cerebrovascular diseases, PTX is recommended at 400–800 mg orally every day; enteric-coated (EC) tablets and sustained-release (SR) tablets are widely used clinically (Ward and Clissold, 1987). Different dosage forms could significantly influence the extent of drug exposure within the body, potentially leading to enhanced efficacy or a higher incidence of adverse reactions, even resulting in diminished efficacy (Cong et al., 2023). However, there is a notable deficiency in the results concerning the pharmacokinetics and relative bioavailability of the SR tablets and the EC tablets. Research indicated that PTX frequently causes gastrointestinal adverse reactions, but food can effectively mitigate these irritation symptoms (Samlaska and Winfield, 1994). Furthermore, previous studies conducted by our research team demonstrated that food intake enhances the relative bioavailability of SR tablets of PTX in beagle dogs (Xu et al., 2023), thus recommending the administration of the drug post-meals. Currently, the specifications for the EC tablets available on the market indicate a dosage of 100 mg per tablet, with a recommended administration of 2–4 tablets. In contrast, the SR tablets contain 400 mg per tablet, with a recommended dosage of one tablet per administration. Consequently, in the context of clinical application, it is essential to investigate whether the differing administration methods of these two preparations may result in variations in the drug’s pharmacokinetics within the body, potentially impacting clinical efficacy. Therefore, an optimized two-period cross-over design was employed in six beagle dogs to explore the relative postprandial relative bioavailability of SR tablets with that of EC tablets of PTX and its primary metabolites M1 and M5.

The measurement of PTX and metabolites' pharmacokinetics were conducted using sensitive, rapid, and robust LC–MS/MS methods. Research on the pharmacokinetics of PTX and its metabolites across various dosage forms may enhance the rational application of this drug and facilitate the development of new pharmaceutical agents.

## Materials and methods

### Chemicals materials

Pentoxifylline sustained-release tablets (Shuanling^®^, 400 mg/tablet, Lot H10970265) and pentoxifylline enteric-coated tablets (Dayi^®^, 100 mg/tablet, Lot H14021860) were gifted by the CSPC Pharmaceutical Group (Shijiazhuang, China). The standard of PTX (purity >99%; E0024848) was gifted by the CSPC Pharmaceutical Group (Shijiazhuang, China).1-(5-Hydroxyethyl)-3,7-dimethylxanthine (M1, purity: 99.9%; 0307-RE-0081) and 1-(3-carboxypropyl)-3,7-dimethylxanthine (M5, purity: 98.4%; 1024-RD-0057) standards were obtained from Cato Research Chemicals Inc. (Guangzhou, China). Phenacetin (purity >99%; 2000601–6012111035-B22052108) was purchased from Bepure Co. Ltd. (Beijing, China). HPLC-grade methanol and acetonitrile were purchased from Fisher Scientific (Waltham, MA, United States). Formic acid and ammonium acetate were obtained from Mreda Technology Inc. (Dallas, TX, United States). Ultrapure water was provided by the Watson and Company (Guangzhou, China).

Six healthy male beagle dogs (10.25–12.60 kg) were provided by Beijing Keyu Biotechnology Co., Ltd. in China (Certificate No. (Jing) 2018-0010). These dogs were not previously exposed to any antibiotic and other drugs during the acclimation or the study periods. They were fasted for 12 h before drug administration and for another 4 h after dosing. Dogs had free access to water during the experiments.

### Instruments and the LC–MS/MS method

The method for determining the concentration of PTX has been thoroughly detailed in prior studies conducted by our research team (Xu et al., 2023) LC–MS/MS analysis of M1 and M5 were performed using an ExionLC™ analytical (UPLC) system (AB Sciex, United States) and an AB Sciex Triple Quad 4500 MD instrument (Applied Biosystems Inc., United States). Data acquisition and quantification were conducted using MultiQuant MD 3.0.3 (AB Sciex, United States). Both instruments were equipped with an electrospray ionization (ESI) source operating in the positive ion mode.

M1 and M5 were analyzed simultaneously using a Kinetex C18 column (100 mm × 2.1 mm, i. d., 1.7 μm, Phenomenex Corporation, MA, United States) at 40°C, with a flow rate of 0.3 mL/min. The mobile phase A was composed of 5 mM ammonium acetate with 0.2% formic acid, and phase B was acetonitrile. The mobile phase B in the LC gradient profile was 20% at beginning and linearly increased to 95% at 1.0 min, maintained at 95% from 1.0 to 2.0 min, returned to 20% at 2.5 min, and maintained at 20% until 4.0 min. The retention times of M1, M5, and phenacetin (IS) were 1.80, 1.50, and 1.94 min, respectively. MS/MS conditions were optimized as follows: source temperature, 450°C; ion spray voltage, 5500 V; curtain gas, 35 psi; collision gas, 8 psi; and dwell time, 100 ms. The ion pairs for positive multiple reaction monitoring (MRM) were m/z 280.9 to 263.2 for M1, m/z 267.3 to 221.4 for M5, and 180.3 →110.2 for IS. The declustering potential was 90, 53, and 70 V for M1, M5, and IS, respectively. The collision energy was set at 15, 24, and 28 eV for M1, M5, and IS, respectively.

### Preparation of the calibration solutions and quality control samples

For the analytes M1 and M5, two individuals prepared the stock solutions for the calibrators (1 mg/mL) and quality controls (1 mg/mL) by weighing, respectively, and stored at −80°C, and the IS stock solution was prepared using methanol and stored at −80°C. M1 and M5 working solutions were obtained by serial dilution in methanol and water (1:1, v/v) to obtain a concentration from 500 ng/mL to 100 μg/mL. The samples for the calibration curve and quality control (QC) in plasma were prepared using blank plasma. The final concentrations of M1 and M5 were 50, 100, 200, 500, 1000, 2000, 5,000, and 10,000 ng/mL after mixing with the blank plasma. QC samples were used at the concentrations of 50, 80, 800, and 8,000 ng/mL. All working solutions, stock solutions, calibration curve samples, and QC samples were stored at 4°C.

### Plasma sample treatment

A measure of 500 μL of methanol containing IS (40 ng/mL phenacetin) was combined with 100 μL of beagle dog plasma in 1.5-mL centrifuge tubes. After vortexing for 3 min and centrifugation at 13,000 rpm for 5 min, 100 µL of the supernatant was mixed with 100 μL of methanol and water (1:1, v/v). The mixture was vortexed for 1 min and centrifuged at 13,000 rpm for 3 min. Finally, 1 μL of the supernatant was injected into the UPLC-MS/MS system for the analysis.

### Method validation

According to the U.S. Food and Drug Administration (FDA) Guidelines on the Bioanalytical Method Validation (2018) (FDA, 2021) and the Chinese Pharmacopoeia (2020), the method for detecting M1 and M5 in the plasma samples of beagle dogs was validated through selectivity, linearity, lower limit of quantification, precision, accuracy, matrix effect, extraction recovery, and stability.

### Experimental design and pharmacokinetic study in beagle dogs

Animal experiments were conducted according to the Regulations of Experimental Animal Administration from the State Committee of Science and Technology of China. All experimental procedures were approved by the Institutional Ethics Committee of the Fourth Hospital of Hebei Medical University (Shijiazhuang, China); the approval number was IACUC-4th Hos Hebmu-2023201.

Six beagle dogs were raised in the Laboratory Animal Center of the Fourth Hospital of Hebei Medical University (Shijiazhuang, China). Beagle dogs were individually kept indoors in pens for 1 month to avoid potential cross-contamination between animals and to adapt to the environment. The beagle dogs had access to water *ad libitum* throughout the study period and were fed once daily with an appropriate ratio of commercial canine feed. Each animal was uniquely identified and acclimatized to the study conditions for at least 1 week. Beagle dogs were housed indoors in climate-controlled facilities, adhering to established laboratory animal care and use guidelines. They were provided with daily opportunities for outdoor exercise and social interaction. Throughout the study, the dogs were observed at least once daily for general health, behavior, and appetite.

A randomized, single-dose, two-period, cross-over study design was used in this study. Six beagle dogs were enrolled and randomly divided into two groups, an SR group and an EC group, with three subjects in each group, with a 5-day washout period. In the SR group, beagle dogs were dosed once orally with 400 mg PTX sustained-release tablets on Day 0. In the EC group, beagle dogs were dosed once orally with 400 mg PTX enteric-coated tablets on Day 0. Tablets were placed on the back of the tongue, and swallowing was stimulated with a small amount of water. Both groups were let to fast for 12 h before PTX administration. All dogs were offered 50 g food ration 15 min before PTX administration. After completing the first cycle of the experiment and the subsequent washout period, the experimental beagle dogs exchanged the group. The experimental protocol for the second cycle remained the same as that of the first cycle.

Blood samples were collected within 30 min (0 min) before tablet administration and then 0.25, 0.5, 0.75, 1, 1.5, 2, 2.5, 3, 4, 6, 8, 10, 12, and 14 h after PTX tablet administration in the SR group. Blood samples were collected 0, 1, 2, 2.67, 3.33, 4, 4.67, 5.33, 6, 7, 8, 9, 10, 12, and 14 h after PTX tablet administration in the EC group. Whole blood (2 mL) was collected into chilled polypropylene tubes containing EDTA-K_2_. Plasma was separated by centrifugation for 10 min at 2,000 rpm, and the plasma was collected and stored at −80°C until analysis.

### Pharmacokinetic analysis

The pharmacokinetic (PK) parameters were analyzed based on a non-compartmental analysis (NCA) using WinNonlin^®^ software (version 8.3.1, Pharsight, United States). PTX and its metabolite M1 and M5 concentrations were obtained from the beagle dogs; plasma drug concentration-time data were fitted to determine the area under the curve (AUC_0→t_ and AUC_0→∞_), elimination half-life (T_1/2_), clearance (CL/F), and the apparent volume of distribution (V_d_/F). The values for the highest plasma drug concentration (C_max_) and the time to reach C_max_ (T_max_) of PTX, M1, and M5 were obtained from the observed data using the concentration–time curve.

### Statistical analysis

Origin 2021^®^ was used to draw plasma concentration–time curves. Analysis of variance (ANOVA) was performed on log-transformed AUC_0→t_, AUC_0→∞_, C_max_, CL/F, and V_d_/F PK parameters. The Wilcoxon pair test was used to assess treatment differences for T_max_ and T_1/2_. The PK evaluation of PTX SR tablets and EC tablets was based on AUC_0→t_, C_max_, and T_max_. The relative bioavailability of PTX SR tablets and EC tablets was evaluated using log-transformed values of AUC_0–t_, AUC_0-∞_, and C_max_. The relative bioavailability of the SR tablets versus EC tablets was estimated as the ratio of geometric LSM of AUC_0–t_, AUC_0-∞_, and C_max_, and associated two-sided 90% CI. Additionally, we utilize the SR/EC ratios of AUC_0→t_ for M1 and M5 relative to PTX, respectively (the AUC_0→t_ ratio of M1/PTX and M5/PTX) to predict and evaluate the bioconversion of drugs within the beagle dogs.

## Results

### Validation of the analysis method

**FIGURE 1:**
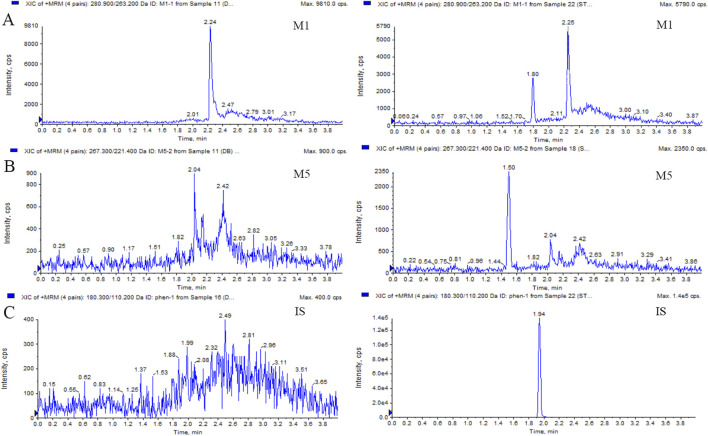
Typical MRM chromatograms of M1 **(A)**, M5 **(B)**, and IS **(C)**: blank plasma sample (left); blank plasma sample spiked with M1 or M5 at 50 ng/mL and IS at 40 ng/mL (right).

**FIGURE 2:**
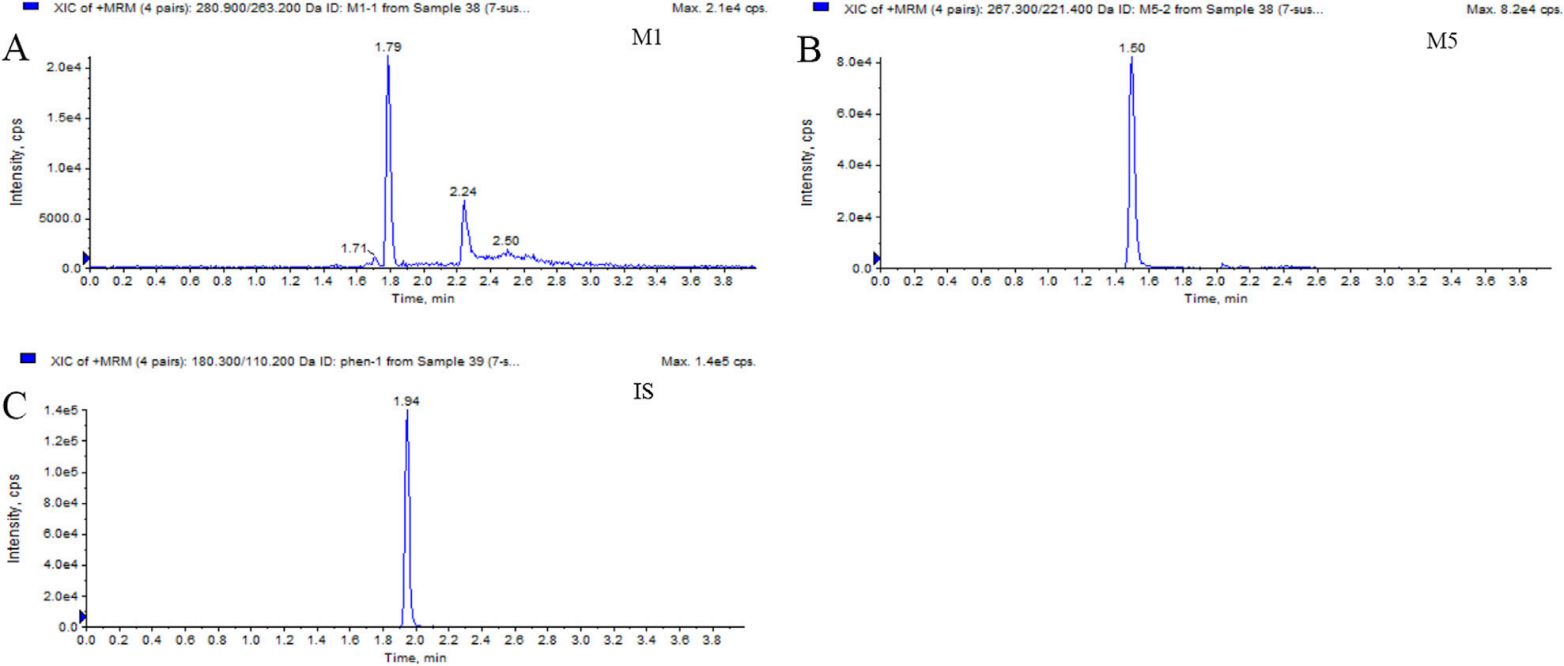
Typical MRM chromatograms of M1 **(A)**, M5 **(B)**, and IS **(C)**: real plasma sample 1.5 h after oral administration of 400 mg PTX SR tablets.

This LC-MS/MS method was robust during the analysis of M1 and M5, with a reliable calibration curve and QC in each analytical batch. The method validation was completed, and the results for M1 and M5 were acceptable within the criterion range (from Supplementary Table S1 to Supplementary Table S3). The selectivity is shown in Figure 1 and Figure 2. No interference was found. The calibration curves of M1 and M5 were linear over the range of 50–10,000 ng/mL in dog plasma, with a correlation coefficient (r^2^) of 0.9946. The lower limit of quantification (LLOQ) was 50 ng/mL (accuracy and precision were 97.36% and 100.2%, respectively). The intra- and inter-batch precision at QC concentrations were <15%, with an accuracy ranging from 89.91% to 105.4%. The extraction recovery of M1 was from 84.77% to 105.6%, and the RSD% values of IS-normalized MFs at M1 concentrations of low-quality control (LQC) and high-quality control (HQC) were 6.79% and 2.56%, respectively. The extraction recovery of M5 was from 98.01% to 98.93%, and the RSD% values of IS-normalized MFs at M5 concentrations of LQC and HQC were 2.55% and 3.01%, respectively. M1 and M5 were stable for 4 h in the plasma at room temperature after three freeze–thaw cycles and for 30 days at −80°C. The post-preparative samples were also stable for 24 h during storage in the autosampler at 4°C. The RSD% of the stability samples was <15%. The 10-fold dilution results indicated that the samples could be linearly diluted (Supplementary Table S4.).

### Pharmacokinetic parameters of PTX in beagle dogs

**FIGURE 3:**
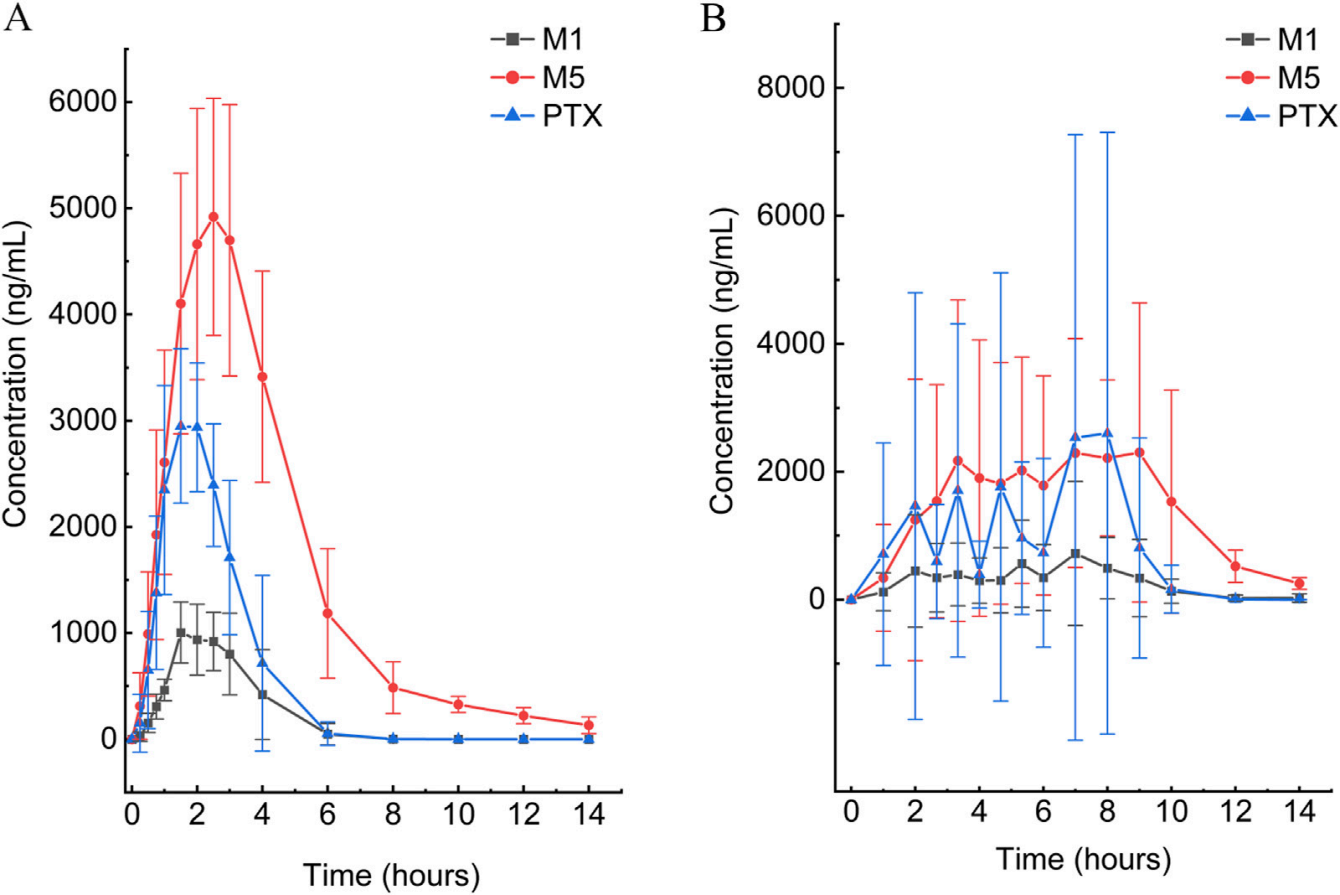
Mean plasma concentration–time curves of PTX, M1, and M5 after a single oral dose of 400 mg in the SR tablet group **(A)** and EC tablet group **(B)** (n = 6).

**FIGURE 4:**
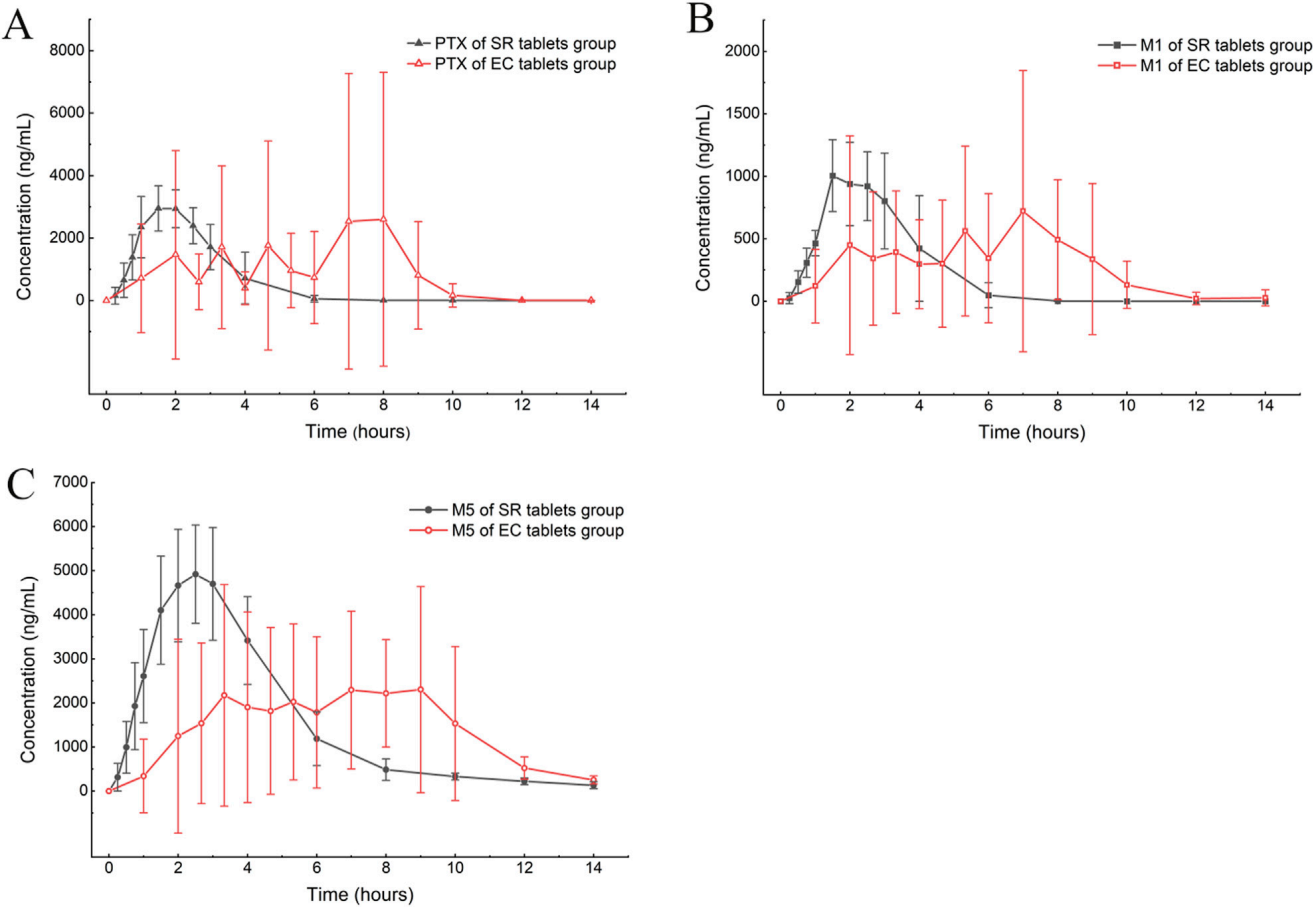
Mean plasma concentration–time curve comparison of PTX **(A)**, M1 **(B)**, and M5 **(C)** after a single oral dose of 400 mg in the SR tablet group and the EC tablet group (n = 6).

According to the experimental design, different commercial tablets from the same batch were assigned to two groups: the SR tablet group and the EC tablet group. The PK profiles of SR tablets and EC tablets are shown in Figure 3. Figure 4 presents a pharmacokinetic comparison of PTX, M1, and M5 between the SR tablet group and the EC tablet group. Major pharmacokinetic parameters of PTX, M1, and M5 based on the non-compartmental approach are listed in Table 1. With the comparison between the SR tablet group and EC tablet group, a significant difference was observed from t_max_ in PTX, M1, and M5 (PTX and M5, *p < 0.05*; M1, *P < 0.01*). Furthermore, a significant difference was observed from C_max_ only in M1(*P < 0.05*). No statistically significant difference was found in other pharmacokinetic parameters (*P > 0.05*).

**TABLE 1:** Pharmacokinetic parameters of PTX, M1, and M5 after a single oral dose of 400 mg in the SR tablet group and the EC tablet group.

Analyte	Parameter	Unit	SR tablet group (mean ± SD, n = 6)	EC tablet group (mean ± SD, n = 6)	SR/EC ratio
PTX	C_max_	ng/mL	3,119.67 ± 776.81	8,207.67 ± 3827.44	0.38
	T_1/2_	h	0.42 ± 0.08	0.38 ± 0.09	1.11
	t_max_	h	1.83 ± 0.52	5.06 ± 2.24*	0.36
	AUC_0-t_	h·ng/mL	8,027.75 ± 2689.75	12,373.97 ± 4935.67	0.65
	AUC_0−∞_	h·ng/mL	8,032.05 ± 2689.93	12,378.47 ± 4932.57	0.65
	CL/F	L/h	54.35 ± 17.06	39.15 ± 21.23	1.39
	V_d_/F	L	32.01 ± 7.26	20.29 ± 8.10	1.58
M1	C_max_	ng/mL	1,020.25 ± 282.66	1,787.33 ± 690.18*	0.57
	T_1/2_	h	0.55 ± 0.18	0.47 ± 0.20	1.17
	t_max_	h	1.83 ± 0.52	5.78 ± 2.73**	0.32
	AUC_0-t_	h·ng/mL	3,067.81 ± 1510.56	3,741.43 ± 1087.08	0.82
	AUC_0−∞_	h·ng/mL	3,074.31 ± 1509.01	3,741.67 ± 1086.86	0.82
	CL/F	L/h	151.98 ± 56.16	115.06 ± 34.19	1.32
	V_d_/F	L	118.80 ± 51.92	77.42 ± 38.98	1.53
M5	C_max_	ng/mL	5,278.17 ± 982.72	5,324.00 ± 1330.50	0.99
	T_1/2_	h	2.03 ± 0.62	1.67 ± 0.61	1.22
	t_max_	h	3.08 ± 0.80	5.78 ± 2.73*	0.53
	AUC_0-t_	h·ng/mL	23,580.51 ± 4218.44	19,897.48 ± 4115.73	1.19
	AUC_0−∞_	h·ng/mL	23,664.01 ± 4218.44	19,914.42 ± 4104.38	1.19
	CL/F	L/h	17.47 ± 3.84	20.95 ± 5.10	0.83
	V_d_/F	L	50.92 ± 18.56	49.73 ± 17.46	1.02

Notes: *P< 0.05 and **P< 0.01 indicate significant differences compared with the SR tablet group.

### Relative bioavailability study of sustained-release tablets and enteric-coated tablets

The relative bioavailability analysis including geometric LSM, ratios, and 90% CI is summarized in Table 2. With regard to the geometric LSM of AUC and C_max_ for SR tablets and EC tablets, the ratios of SR/EC of PTX, M1, and M5 were 67.62% (90% CI, 50.49%–90.55%), 78.18% (90% CI, 54.15%–112.88%), and 119.11% (90% CI, 99.62%–142.41%) for AUC_0-t_; 67.62% (90% CI, 50.50%–90.55%), 78.36% (90% CI, 54.48%–112.72%), and 119.39% (90% CI, 100.03%–142.50%) for AUC_0−∞_; and 54.36% (90% CI, 36.63%–80.67%), 58.80% (90% CI, 40.84%–84.66%), and 100.51% (90% CI, 89.50%–112.88%) for C_max_, respectively, suggesting a very close relationship between PTX and M1, while a distinct difference was evident when comparing M5 to these two. Table 3 presented the SR/EC ratios of the AUC_0→t_ for M1 and M5 relative to PTX; the M1/PTX values of AUC_0→t_ between the SR tablet group and EC tablet group were 0.37 and 0.36, the SR/EC ratio for M1/PTX was 1.03. The M5/PTX values of AUC_0→t_ between the SR tablet group and EC tablet group were 3.09 and 1.91, and the SR/EC ratio for M5/PTX was 1.62; a significant difference about SR/EC ratio was observed in M5/PTX between two groups (*p < 0.05*).

**TABLE 2:** Geometric least square mean values, ratios, and 90% CIs for pharmacokinetic parameters of PTX, M1, and M5 between the SR tablet group and the EC tablet group.

Analyte	Parameter	Geometric LSM		Ratio of SR/EC (%)	90% CI (%)
		SR	EC		
PTX	AUC_0-t_ (h·ng/mL)	7,680.78	11,359.15	67.62	50.49–90.55
	AUC_0−∞_ (h·ng/mL)	7,681.21	11,359.20	67.62	50.50–90.55
	C_max_ (ng/mL)	3,037.27	5,587.17	54.36	36.63–80.67
M1	AUC_0-t_ (h·ng/mL)	2,820.33	3,607.53	78.18	54.15–112.88
	AUC_0−∞_ (h·ng/mL)	2,827.20	3,607.83	78.36	54.48–112.72
	C_max_ (ng/mL)	991.64	1,686.53	58.80	40.84–84.66
M5	AUC_0-t_ (h·ng/mL)	23,228.41	19,501.78	119.11	99.62–142.41
	AUC_0−∞_ (h·ng/mL)	23,305.85	19,520.27	119.39	100.03–142.50
	C_max_ (ng/mL)	5,203.28	5,176.73	100.51	89.50–112.88

**TABLE 3:** AUC_0-t_ ratios of M1 and M5 to PTX in beagle dogs.

	SR tablet group (mean ± SD, n = 6)	EC tablet group (mean ± SD, n = 6)	SR/EC ratio
M1/PTX	0.37 ± 0.07	0.36 ± 0.19	1.03
M5/PTX	3.09 ± 0.66	1.91 ± 0.98	1.62*

Notes: *P< 0.05 indicates significant differences, compared with M1/PTX.

## Discussion

Pharmacokinetic results indicated that M5 exhibited the highest exposure level, while M1 demonstrated the lowest in both the SR and EC tablet groups. For the prototype drug PTX, rapid absorption was observed in the SR tablet group (t_max_ was 1.83 h), accompanied by a quick elimination (T_1/2_ was 0.42 h). In contrast, the EC tablet group exhibited a significantly prolonged absorption process (t_max_ was 5.06 h), with a higher degree of exposure in the body compared to the SR tablet group (the AUC ratio of SR/EC was 0.65). Similar results were observed in M1, which exhibited behavior analogous to that of PTX. The difference in C_max_ was more pronounced for M1 compared to PTX. There was a statistically significant difference (*p < 0.05*) in the C_max_ value of M1 between the SR tablet group and the EC tablet group. It was important to note that the oxidative metabolite M5 and the reduced metabolite M1 exhibited distinct differences in their pharmacokinetic characteristics. Both the SR tablet group and the EC tablet group demonstrated a significant prolongation of T_1/2_ of M5, with values of 2.03 h for the SR tablet group and 1.67 h for the EC tablet group compared to PTX and M1. Furthermore, in the EC tablet group, t_max_ of M5 was found to be longer than that observed in the SR tablet group (3.08 h vs. 5.78 h). Additionally, the exposure levels of M1 and M5 in the two different formulation exhibited contrasting patterns, and the AUC ratio of M5 between the two groups (SR/EC) was 1.19, which differs from the AUC ratios of PTX at 0.65 and M1 at 0.82.

In the relative bioavailability study, PTX and M1 performed a high relative bioavailability of the EC tablets compared to the SR tablets, the AUC ratios of SR/EC were 67.62% and 78.18%, respectively. Conversely, in M5, the SR tablets exhibited a higher relative bioavailability compared to the EC tablets with 119.11% of the AUC ratio of SR/EC. Additionally, we calculated the ratio of the AUC of metabolites M1 and M5 to the AUC of PTX for predicting conversion rates of PTX into metabolites M1 and M5 across different preparations. The results indicated that there was no significant difference in the bioconversion rate of M1 between the two preparations, with a conversion rate ratio of 1.02. In contrast, the conversion rate of M5 demonstrated a significant difference (*P< 0.05*) between the two preparations, with a ratio of 1.62. These findings suggested that, compared to EC tablets, SR tablets might exhibit a higher degree of biotransformation within the body. We supposed that the reasons for this difference might be attributed to variations in the drug’s release process and influenced by the formulation absorption. Previous research had indicated that a significant difference between M1 and M5 lies in the conversion of PTX to M1, which occurs primarily in erythrocytes and reversible in red blood cells. In contrast, M5 was metabolized exclusively by the liver (Nicklasson et al., 2002). Studies involving healthy subjects with liver cirrhosis and renal dysfunction demonstrated that the ratio of M1 to the prototype drug PTX was consistent with the findings observed in healthy subjects (Rames et al., 1990; Paap et al., 1996). Furthermore, a comparison of the AUC ratios of each metabolite and PTX, following oral administration, revealed that the AUC ratio of the oxidative metabolite M5 was elevated, while the AUC ratio of the total concentration of M1 was relatively low. This finding further validated the differences observed between the two metabolites (Vlase et al., 2010). It is noteworthy that differences in the conversion rates of the two metabolites were observed across the various formulations. Specifically, the conversion rate of M5 in SR tablets was significantly higher than that of EC tablets, likely due to the prolonged, smoother, and more complete release characteristics of SR tablets. The sustained-release matrix material utilized in this study’s formulation is Kollidon^®^ SR. Research indicated that this matrix material is less susceptible to food effects, thereby enhancing patient medication compliance (Song et al., 2016). Furthermore, Kollidon^®^ SR had been shown to improve the mechanical properties of tablets, resulting in increased hardness and reduced friability while also maintaining the structural stability of the dosage form until the drug is fully dissolved (Sakr et al., 2011). This evidence might further elucidate why the SR tablets exhibited superior *in vivo* performance compared to the EC tablets, contributing to a more consistent release profile. In contrast, this difference was not evident in the two dosage forms of M1. This discrepancy might be attributed to its unique metabolic process, which allows for conversion into the prototype drug (Nicklasson et al., 2002). This process enables PTX and M1 to engage in a dynamic mutual conversion within the body, thereby masking the variations associated with dosage form factors. This observation further suggested that SR tablets exhibit superior release effects in the body. Furthermore, changes resulting from the saturation of elimination enzymes and transporters might also occur when a drug is released very rapidly, such as in the case of immediate-release formulations (Seibel et al., 2023).

Additionally, significant inter-individual differences and irregularities in drug absorption and metabolism were observed in the EC tablet group after meal, which aligns with the well-documented patterns of gastric emptying observed with other EC tablets, as reported in the FDA labeling for Depakote, EC-Naprosyn, and Voltaren (Cameron et al., 2020). These variations were manifested by the emergence of multiple absorption peaks in the average drug concentration-time curve, as well as notable differences in PK parameters among individuals, especially the significant prolongation of t_max_ for PTX, M1, and M5. Since delayed absorption and multiple release of EC tablets in beagle dogs were not observed in fasting studies (data not published due to concerns regarding drug safety), we concluded that the variation in EC tablets is primarily attributed to food effects. Food intake altered the composition and properties of digestive juices, including various digestive enzymes, pH, viscosity, and osmotic pressure, and t_max_ of EC tablets, which is significantly influenced by gastric emptying (US Food and Drug Administration, 2002). These changes might subsequently affect the absorption, distribution, metabolism, and excretion of drugs (Singh, 1999). Numerous studies had demonstrated that formulations exhibiting pH-dependent release properties, such as enteric formulations, possess a significant potential for interactions with food, characterized by the presence of sizable, indigestible solid particles. The passage of these particles through the pylorus typically occurs during the stage III migrating motor complex. However, food intake disrupts the migrating motor complexes, which can result in a delay in the transit of enteric-coated preparations from the stomach to the duodenum (Paap et al., 1996; Zhang et al., 2020). Moreover, food intake influenced the disintegration of immediate-release preparations in the stomach, leading to a reduced disintegration rate and, consequently, delayed drug absorption in the body (Radwan et al., 2014). The variation in peak times among the tested beagles is the primary reason for the observed ‘double peak’ in the mean pharmacokinetic curve depicted in the figure (Cameron et al., 2020).

Despite the widespread use of various dosage forms of PTX among the Chinese population, there remains a lack of studies investigating the *in vivo* pharmacokinetic differences between the SR tablets and the EC tablets. The results of this study indicated that under postprandial administration conditions, the SR tablets demonstrated more stable and controllable release characteristics compared to the EC tablets. Furthermore, the biotransformation level of the SR tablets in the body appeared to be superior to that of the EC tablets, suggesting that the SR tablets might be more effectively converted into the active form of PTX. Additionally, no significant adverse reactions were observed in any of the experimental subjects, indicating that both preparations possess a favorable safety profile. Based on these findings and considering improved medication compliance, we proposed that SR tablets may hold greater potential for clinical applications.

Certainly, this study acknowledges certain limitations. It is a bioequivalence study conducted in beagle dogs, and there is currently a lack of data regarding the two drugs in humans. A larger sample size may be required to enhance the validation of the results obtained in this study. Additionally, this study did not assess the safety and efficacy of long-term use of the two dosage forms. Future research should include further pharmacokinetic and pharmacodynamic studies in both healthy individuals and patients.

In summary, no significant statistical differences were observed in the total exposure levels (AUC) of PTX, M1, and M5 between SR and EC tablets. However, SR tablets exhibited smaller inter-individual variability, following meals, indicating a more stable and controllable drug release process. Furthermore, findings in beagles suggest that the conversion of the drug to its active metabolites might be more complete in sustained-release tablets. Therefore, in comparison to EC tablets, SR tablets could offer broader application prospects; however, further human studies are necessary to be proved.

## Conclusion

Pharmacokinetic study results in beagle dogs indicated that PTX and its reduced metabolite M1 exhibited similar pharmacokinetic characteristics, whereas the oxidative metabolite M5 displayed certain differences. The exposure levels of PTX and M1 in the SR tablet group were lower than those in the EC tablet group. Conversely, the exposure level of M5 in the SR tablet group was higher than that in the EC tablet group. However, this difference in exposure did not yield a statistically significant difference in AUC. The EC tablet group demonstrated notable inter-individual differences and irregular drug absorption, following meals. Moreover, it seems that SR tablets were converted to active metabolites to a greater extent within the beagle dog compared to EC tablets. Consequently, the SR tablets appeared to provide a more stable and controllable therapeutic effect in beagle dogs.

## Data Availability

The original contributions presented in the study are included in the article/Supplementary Material; further inquiries can be directed to the corresponding author.
