# Supplementary material for: Evaluation of pharmacokinetics and relative bioavailability of pentoxifylline and its metabolite in beagle dogs following different formulations
Source: Front Pharmacol. 2024 Nov 20;15:1488076. doi: 10.3389/fphar.2024.1488076 (PMC11614658; doi:10.3389/fphar.2024.1488076)
Supplement: Supplementary file 1 [file Table1.DOCX]

| Analyte | Spiked Conc.  (ng/mL) | Intra-batch (n=5) | | | | Inter-batch (n=15) | | |
| --- | --- | --- | --- | --- | --- | --- | --- | --- |
|  |  | Mean±  SD (ng/mL) | Precision  (RSD%) | Accuracy  (RE%) | Mean±  SD (ng/mL) | | Precision  (RSD%) | Accuracy  (RE%) |
| M1 | 50 | 48.67±4.941 | 10.2 | 97.36 | 48.98±4.192 | | 8.55 | 98.01 |
|  | 80 | 71.95±2.703 | 3.75 | 89.91 | 77.73±5.506 | | 7.07 | 97.20 |
|  | 800 | 769.2±53.75 | 6.75 | 99.52 | 821.8±38.08 | | 4.63 | 103.3 |
|  | 8000 | 7672±307.2 | 4.00 | 95.94 | 7569±229.0 | | 3.02 | 94.62 |
| M5 | 50 | 48.41±4.310 | 8.91 | 96.84 | 50.46±4.522 | | 8.96 | 101.6 |
|  | 80 | 77.00±2.477 | 3.21 | 96.37 | 81.85±5.315 | | 6.48 | 102.7 |
|  | 800 | 824.9±34.07 | 4.13 | 103.2 | 847.6±36.83 | | 4.34 | 105.4 |
|  | 8000 | 8174±236.7 | 2.90 | 102.5 | 8088±206.0 | | 2.55 | 101.3 |

Table S1. Precision and accuracy data of M1 and M5 in beagle dog plasma

Table S2. Matrix effect and extraction recovery of M1 and M5 in beagle dog plasma.

| Analyte | Spiked Conc.  (ng/mL) | Extraction Recovery (n=5) | | IS normalized matrix effect (n=6) | |
| --- | --- | --- | --- | --- | --- |
|  |  | Mean±SD(%) | RSD (%) | Mean±SD(%) | RSD (%) |
| M1 | 80 | 84.77±8.00 | 9.43 | 120.2±8.17 | 6.79 |
|  | 800 | 87.77±2.24 | 2.55 | / | / |
|  | 8000 | 105.6±4.45 | 4.21 | 122.2±3.13 | 2.56 |
| M5 | 80 | 98.01±5.24 | 5.34 | 117.4±3.00 | 2.55 |
|  | 800 | 98.93±3.36 | 3.39 | / | / |
|  | 8000 | 98.22±6.59 | 6.71 | 115.8±3.46 | 3.01 |

| Analyte | Conc.  (ng/mL) | Room Temperature for 4 h | Automatic Sampler at 4 °C for 24 h | 3 Freeze-Thaw Cycles, -80 °C to Room Temperature | Long-term Stability -80 °C for 4 Weeks |
| --- | --- | --- | --- | --- | --- |
| M1 | 50 | 93.93±8.17 | 94.52±3.61 | 100.03±11.05 | 96.53±6.63 |
|  | 80 | 98.27±3.78 | 99.96±9.77 | 101.9±9.33 | 99.69±3.62 |
|  | 800 | 112.2±2.17 | 109.2±5.93 | 105.8±4.82 | 102.8±1.79 |
|  | 8000 | 104.5±2.92 | 93.26±10.64 | 92.40±6.68 | 95.53±4.68 |
| M5 | 50 | 103.6±6.30 | 89.40±6.38 | 98.50±6.08 | 107.4±9.65 |
|  | 80 | 104.8±5.01 | 99.27±3.43 | 104.1±6.53 | 106.5±5.42 |
|  | 800 | 112.0±1.12 | 106.2±1.94 | 110.9±2.65 | 103.8±1.64 |
|  | 8000 | 106.8±2.79 | 93.67±2.58 | 102.0±1.01 | 99.1±2.43 |

Table S3. Stability of M1 and M5 in beagle dog plasma under various storage conditions (mean ± SD, n=5, %).

Table S4. Evaluation of the dilution integrity of M1 and M5 in beagle dog plasma (n=5).

| Analyte | Dilution fold | Accuracy (%)  Mean±SD | RSD (%) |
| --- | --- | --- | --- |
| M1 | 1:10 | 95.04±8.29 | 8.72 |
| M5 | 1:10 | 95.00±6.60 | 6.95 |
